# Andronov–Hopf and Neimark–Sacker bifurcations in time-delay differential equations and difference equations with applications to models for diseases and animal populations

**DOI:** 10.1186/s13662-020-02646-5

**Published:** 2020-04-29

**Authors:** Rachadawan Darlai, Elvin J. Moore, Sanoe Koonprasert

**Affiliations:** 1 Faculty of Science, Energy and Environment, King Mongkuts University of Technology North Bangkok (Rayong Campus), Rayong, Thailand; 2 Department of Mathematics, King Mongkuts University of Technology North Bangkok, Bangkok, Thailand; 3 Centre of Excellence in Mathematics, CHE, Bangkok, Thailand

**Keywords:** 05C69, 05C70, 05C76, Andronov–Hopf bifurcation, Neimark–Sacker bifurcation, Time delay, Asymptotic stability, Limit cycles, HIV model, Extended logistic growth model

## Abstract

In many areas, researchers might think that a differential equation model is required, but one might be forced to use an approximate difference equation model if data is only available at discrete points in time. In this paper, a detailed comparison is given of the behavior of continuous and discrete models for two representative time-delay models, namely a model for HIV and an extended logistic growth model. For each model, there are seven different time-delay versions because there are seven different positions to include time delays. For the seven different time-delay versions of each model, proofs are given of necessary and sufficient conditions for the existence and stability of equilibrium points and for the existence of Andronov–Hopf bifurcations in the differential equations and Neimark–Sacker bifurcations in the difference equations. We show that only five of the seven time-delay versions have bifurcations and that all bifurcation versions have supercritical limit cycles with one having a repelling cycle and four having attracting cycles. Numerical simulations are used to illustrate the analytical results and to show that critical times for Neimark–Sacker bifurcations are less than critical times for Andronov–Hopf bifurcations but converge to them as the time step of the discretization tends to zero.

## Introduction

In recent years, time-delay differential equation and difference equation models have been studied by many authors (see, e.g., [1–16]) as they are useful tools for modeling a wide variety of systems in areas including traditional areas such as physics and engineering and newer areas such as disease transmission, medical research, optimal drug treatment, bioeconomics, agriculture, finance, insurance, and environmental protection. In many of these time-delay models, bifurcations occur as values of parameters are changed. For example, Andronov–Hopf bifurcations can occur in differential equation systems and Neimark–Sacker bifurcations can occur in difference equation systems at critical values of time delays. In some cases, the changes in system parameters can also lead to chaotic-type solutions (see, e.g. [17, 18]).

In many applications, the preferred model is a differential equation model. However, as the growth rate of diseases (such as HIV) or other kinds of populations (such as fish or animal populations) can be a slow process or the collection of data may often only be carried out at regular intervals such as a month or a year, it is often only possible to construct difference equation models. One method that is often used to construct a difference equation model is to use a first-order Euler method to approximate the differential equation model [1, 2, 19–22]. This method is also often used to solve Itó stochastic differential equations as, for example, in the Euler–Mayurama method [1, 23].

As stated above, an important property of many time-delay differential equation models is that they have Andronov–Hopf bifurcations and an important property of many time-delay difference equation models is that they have Neimark–Sacker bifurcations [24]. Because difference equation models are often used to approximate differential equation models, we believe that it is important to study the relationship between the bifurcations in the differential equations and the approximating difference equations.

In this paper, we analyze the bifurcation properties of one-dimensional time-delay differential equation models and the corresponding discrete models obtained by the forward Euler approximation. As examples, we consider time-delay versions of two systems. The first system is a model discussed by Roberts and Saha [25] and Ding et al. [26] for transmission of HIV in a human population. This model includes the effects of vertical HIV transmission from mother to baby, the effects of births and deaths and of treatment by antivirals. The second system is an extended logistic growth model (ELM) that has been applied to forecasting short lifecycle products and services by Trappey and Wu [27], and to growth of single-species populations by He et al. [1] and Sakanoue [28].

For both the HIV and the ELM models, there are seven different versions of time-delay models that can be created from the original models. For each model, we first investigate the properties of the seven different versions of differential equation models and prove conditions for the existence and stability of equilibrium points and for the existence of Andronov–Hopf bifurcations at critical values of the time delays. We then investigate the properties of discretized versions of the models and prove conditions for the existence and stability of equilibrium points and for the existence of Neimark–Sacker bifurcations at critical values of the time delays. We show that both differential equation and difference equation models may have bifurcations from both disease-free and endemic equilibrium points. For the disease-free equilibrium, four of the seven different versions satisfy the bifurcation conditions, and for the endemic equilibrium five of the seven versions have bifurcations. For the bifurcations from the endemic equilibrium points, we show that for each version with bifurcations the critical delay times for Neimark–Sacker bifurcations are less than the critical times for Andronov–Hopf bifurcations but converge to them as the time step of the discretization in the discretized model tends to zero. Numerical simulations are presented for a range of parameter values to illustrate the analytical results. Applications of the results are given to the modeling of HIV transmission and to the modeling of populations.

## Differential equation models

In this section, we describe the differential equation models for HIV and extended logistic growth (ELM) that we study in this paper.

### HIV models

Many researchers have developed mathematical models to study the transmission of HIV at either the CD4+ T-cell level (see, e.g., [3, 4, 7, 29–33]) or at the population level (see, e.g., [25, 26, 34, 35]). In the present research, we study a model for transmission of HIV at the population level originally proposed by Roberts and Saha [25] as a general epidemic model, and later developed by Ding et al. [26] as a stochastic differential equation model for the progression to AIDS in a population infected with HIV. The nonlinear, logistic-type, differential equation model discussed by Ding et al. is as follows (see also [35]):

1 $$ \frac{dx(t)}{dt}=(p-1)Bx(t)+(\beta C -\alpha )x(t) \bigl(1-x(t)\bigr), $$

where $x(t)$ is the proportion of the total population that is infected by HIV at time *t*, *p* ($0< p<1$) is the vertical transmission probability (the fraction of babies born with HIV infection), *B* is the birth rate for the population, *β* is the transmission rate on contact between an infected and an uninfected individual, *C* is the contact rate between infected and uninfected individuals, and *α* is the increase of the death rate due to the HIV infection.

The development of antiretroviral therapy using reverse transcriptase inhibitors (RTI) and protease inhibitors (PI) has been shown to be an effective method of controlling the spread of HIV by depressing the level of virus in an HIV+ person below a detectable level [29–33, 36] and to effectively stop transmission of HIV from an HIV+ person to an uninfected person [37–42]. In the present model, we assume that the effects of both the RTI and the PI can be included in the model in (1) as factors reducing the value of *β* (the rate of infection on contact) and *p* (the vertical transmission probability). Following Darlai et al. [35], we assume that

2 $$ \beta = (1-n_{\mathrm{av}})\beta _{0},\qquad p = (1-n_{\mathrm{av}})p_{0}, $$

where $n_{\mathrm{av}}$ is an antiretroviral therapy factor ($0 \leq n_{\mathrm{av}} < 1$) and $\beta _{0}$ and $p_{0}$ are, respectively, the infection rate of a susceptible person and the vertical transmission probability in the absence of antiretroviral therapy.

For simplicity, we rewrite (1) as

3 $$ \frac{dx(t)}{dt}=-\delta x(t)+\varepsilon x(t) \bigl(1-x(t) \bigr), $$

where $\delta = (1-p)B > 0$ and $\varepsilon = \beta C -\alpha > 0$. Equation (3) is the well-known logistic equation model with an added death rate term $-\delta x(t)$.

### Extended logistic growth models

As stated previously, extended logistic growth models have been applied to forecasting short lifecycle products and services by Trappey and Wu [27], and to growth of single-species populations by Sakanoue [28].

In this paper, we study the extended logistic growth model discussed by He et al. [1]:

4 $$ \frac{dx(t)}{dt} =-rx(t)+\beta x(t)\bigl[1-\bigl(x(t)/K \bigr)^{\gamma }\bigr], $$

where $x(t)$ is the population at time *t*, and *r* is the natural death rate of the population at low population levels. The parameters *β*, *γ*, *K* are positive parameters, where *β* is a natural birth rate at low population levels, *K* is the “carrying capacity” of the system, and the factor $(x(t)/K)^{\gamma }$ is the rate at which the birth rate decreases as $x(t) \rightarrow K$. This reduction in the birth rate could be due, for example, to factors such as overcrowding or limits in the available food supply.

## Time-delay differential equation models

In this paper, we extend the models in Eqs. (3) and (4) by introducing time delays into the models. As shown in Table 1, there are seven time-delay versions of the two differential equation models. We use code “n” for no time delay and “d” for time delay. Then, for example, “dnd” means time delays in positions 1 and 3 and no time delay in position 2.

**Table 1:** Time-delay differential equation models

Version	HIV
1: dnn	$\frac{dx(t)}{dt}=-\delta x(t-\tau )+\varepsilon x(t)[1-x(t)]$
2: ndn	$\frac{dx(t)}{dt}=-\delta x(t)+\varepsilon x(t-\tau )[1-x(t)]$
3: nnd	$\frac{dx(t)}{dt}=-\delta x(t)+\varepsilon x(t)[1-x(t-\tau )]$
4: ddn	$\frac{dx(t)}{dt} =-\delta x(t-\tau )+\varepsilon x(t-\tau )[1-x(t)]$
5: dnd	$\frac{dx(t)}{dt}=-\delta x(t-\tau )+\varepsilon x(t)[1-x(t-\tau )]$
6: ndd	$\frac{dx(t)}{dt}=-\delta x(t)+\varepsilon x(t-\tau )[1-x(t-\tau )]$
7: ddd	$\frac{dx(t)}{dt}=-\delta x(t-\tau )+\varepsilon x(t-\tau )[1-x(t-\tau )]$

Version	ELM
1: dnn	$\frac{dx(t)}{dt} = -rx(t-\tau )+\beta x(t)[1-(x(t)/K)^{\gamma }] $
2: ndn	$\frac{dx(t)}{dt}=-rx(t)+\beta x(t-\tau )[1-(x(t)/K)^{\gamma }]$
3: nnd	$\frac{dx(t)}{dt} =-rx(t)+\beta x(t)[1-(x(t-\tau )/K)^{\gamma }]$
4: ddn	$\frac{dx(t)}{dt} = -rx(t-\tau )+\beta x(t-\tau )[1-(x(t)/K)^{\gamma }] $
5: dnd	$\frac{dx(t)}{dt}=-rx(t-\tau )+\beta x(t)[1-(x(t-\tau )/K)^{\gamma }]$
6: ndd	$\frac{dx(t)}{dt}=-rx(t)+\beta x(t-\tau )[1-(x(t-\tau )/K)^{\gamma }] $
7: ddd	$\frac{dx(t)}{dt} =-rx(t-\tau )+\beta x(t-\tau )[1-[x(t-\tau )/K)^{\gamma }] $

Darlai et al. [35] have studied the Andronov–Hopf and Neimark–Sacker bifurcations in HIV3:nnd and HIV7:ddd, and He et al. [1] have studied the Neimark–Sacker bifurcations in ELM6:ndd.

## Time-delay difference equation models

Using the first-order Euler method, we discretize the independent variable *t* with a step size *h*, and replace a first-order system of differential equations of the form

5 $$ \begin{aligned} &\frac{dx}{dt} = f\bigl(t,x(t)\bigr), \quad t \in [t_{0},T],\qquad x(t_{0}) = x_{0} \quad \mbox{by} \\ &w_{n+1} = w_{n} + h f(t_{n},w_{n}),\qquad w_{0} = x_{0}, \end{aligned} $$

where $t_{n} = t_{0} +nh$, $n=0,1,2,\ldots $ , and $w_{n} = x(t_{n})$. The HIV and ELM difference equation models for zero time delays can then be written in the form

6 $$ \begin{aligned} &\mbox{HIV:} \quad w_{n+1} = w_{n} -\delta h w_{n} + \varepsilon h w_{n}(1-w_{n}), \\ &\mbox{ELM:} \quad w_{n+1} = w_{n} -r h w_{n} + \beta h w_{n}[1-(w_{n}/K)^{\gamma }]. \end{aligned} $$

### Note

As in all difference equation approximations to differential equations, it is necessary to check that the approximation is a numerically stable approximation to the original differential equation. It is well known (see, e.g., [43]) that the forward Euler approximation can be numerically unstable unless an upper limit is placed on the step size *h*. It is therefore necessary to check that a Neimark–Sacker bifurcation corresponds to an Andronov–Hopf bifurcation of the original differential equation model and that it is not a bifurcation arising from the instability of the forward-Euler method.

To obtain the first-order Euler approximations to the seven HIV and ELM time-delay differential equations in Table 1, we assume that the time delay *τ* is divided into *m* equal intervals. Then the step size is $h=h_{m}\tau $, where $h_{m}=1/m$, and we obtain the seven difference equations in Table 2. It can be seen that each of these Euler approximation equations are difference equations of order $m+1$.

**Table 2:** Discrete time-delay equations for HIV and ELM

Version	HIV
1: dnn	$w_{n+1} = w_{n}-\delta h_{m}\tau w_{n-m} +\varepsilon h_{m} \tau w_{n}(1-w_{n})$
2: ndn	$w_{n+1} = w_{n}-\delta h_{m}\tau w_{n} +\varepsilon h_{m} \tau w_{n-m}(1-w_{n}) $
3: nnd	$w_{n+1} = w_{n}-\delta h_{m}\tau w_{n} +\varepsilon h_{m} \tau w_{n}(1-w_{n-m})$
4: ddn	$w_{n+1} = w_{n}-\delta h_{m}\tau w_{n-m} +\varepsilon h_{m} \tau w_{n-m}(1-w_{n})$
5: dnd	$w_{n+1} = w_{n}-\delta h_{m}\tau w_{n-m} +\varepsilon h_{m} \tau w_{n}(1-w_{n-m}) $
6: ndd	$w_{n+1} = w_{n}-\delta h_{m}\tau w_{n} +\varepsilon h_{m} \tau w_{n-m}(1-w_{n-m})$
7: ddd	$w_{n+1} = w_{n}-\delta h_{m}\tau w_{n-m} +\varepsilon h_{m} \tau w_{n-m}(1-w_{n-m}) $

Version	ELM
1: dnn	$w_{n+1} = w_{n}-r h_{m}\tau w_{n-m} +\beta h_{m} \tau w_{n}[1-(w_{n}/K)^{\gamma }]$
2: ndn	$w_{n+1} = w_{n}-r h_{m}\tau w_{n} +\beta h_{m} \tau w_{n-m}[1-(w_{n}/K)^{\gamma }]$
3: nnd	$w_{n+1} = w_{n}-r h_{m}\tau w_{n} +\beta h_{m} \tau w_{n}[1-(w_{n-m}/K)^{\gamma }]$
4: ddn	$w_{n+1} = w_{n}-r h_{m}\tau w_{n-m} +\beta h_{m} \tau w_{n-m}[1-(w_{n}/K)^{\gamma }]$
5: dnd	$w_{n+1} = w_{n}-r h_{m}\tau w_{n-m} +\beta h_{m} \tau w_{n}[1-(w_{n-m}/K)^{\gamma }]$
6: ndd	$w_{n+1} = w_{n}-r h_{m}\tau w_{n} +\beta h_{m} \tau w_{n-m}[1-(w_{n-m}/K)^{\gamma }] $
7: ddd	$w_{n+1} = w_{n}-r h_{m}\tau w_{n-m} +\beta h_{m} \tau w_{n-m}[1-(w_{n-m}/K)^{\gamma }]$

## Equilibrium points, stability and Andronov–Hopf bifurcations of differential equation models

### Equilibrium points

For each model, the equilibrium points $x^{*}$ for the differential equations (3) and (4), and the seven time-delay differential equation versions in Table 1 are the same and are obtained by setting $\frac{dx}{dt} = 0$. For each model, there is a trivial equilibrium point $x^{*} = 0$ and an endemic equilibrium point which exists only if $x^{*} > 0$. The equilibrium points for the models are

7 $$ \begin{aligned} &\mbox{HIV:}\quad x^{*}= 0, \qquad x^{*} = 1 - \frac{\delta }{\varepsilon } = 1-\frac{1}{R_{0}}, \qquad R_{0}=\frac{\varepsilon }{\delta }; \\ &\mbox{ELM:}\quad x^{*} = 0, \qquad x^{*} = K (1- \frac{r}{\beta } )^{\frac{1}{\gamma }} = K (1- \frac{1}{R_{0}} )^{\frac{1}{\gamma }}, \qquad R_{0} = \frac{\beta }{r} . \end{aligned} $$

For both models, the endemic equilbrium exists only if $R_{0} > 1$.

### Local asymptotic stability

The conditions for local asymptotic stability of the equilibrium points can be obtained by using a standard approach, such as the next-generation method [44], or by checking the eigenvalues of the linearized system at the equilibrium points [45]. In this case, it is easy to check the eigenvalues of the linearized equations about the equilibrium points.

We can obtain the linearized versions of the delay equations in Table 1 by defining perturbations $y(t) = x(t) - x^{*}$ and $y(t-\tau ) = x(t-\tau )-x^{*}$. The linearized versions can then be written in the standard form

8 $$ \frac{dy}{dt} = \rho y(t) - \eta y(t-\tau ), $$

where the *ρ* and *η* values for the linearized equations at the disease-free and endemic equilibrium points are shown in Table 3.

**Table 3:** Values of *ρ* and *η* in linearized delay equations at equilibrium points

Version	HIV: $R_{0} =\frac{\varepsilon }{\delta }$				ELM: *Λ* = *β* − *r*, $R_{0} = \frac{\beta }{r}$			
	Disease-free		Endemic		Disease-free		Endemic	
	*ρ*	*η*	*ρ*	*η*	*ρ*	*η*	*ρ*	*η*
1: dnn	*ε*	*δ*	2*δ* − *ε*	*δ*	*β*	*r*	*r* − *γΛ*	*r*
2: ndn	−*δ*	−*ε*	−*ε*	−*δ*	−*r*	−*β*	−*r* − *γΛ*	−*r*
3: nnd	*ε* − *δ*	0	0	*ε* − *δ*	*β* − *r*	0	0	*γΛ*
4: ddn	0	*δ* − *ε*	−*ε* + *δ*	0	0	*r* − *β*	−*γΛ*	0
5: dnd	*ε*	*δ*	*δ*	*ε*	*β*	*r*	*r*	*r* + *γΛ*
6: ndd	−*δ*	−*ε*	−*δ*	*ε* − 2*δ*	−*r*	−*β*	−*r*	−*r* + *γΛ*
7: ddd	0	*δ* − *ε*	0	*ε* − *δ*	0	*r* − *β*	0	*γΛ*

As usual, we assume a trial solution $y(t)=e^{\lambda t}$. The characteristic equation from the trial solution is then

9 $$ \lambda = \rho - \eta e^{-\lambda \tau }. $$

Then, if the real parts of all eigenvalues *λ* of (9) are negative, the general solution $y(t) \rightarrow 0$ as $t \rightarrow \infty $ and the equilibrium point $x^{*}$ is locally asymptotically stable.

*Zero time delay*. For both disease-free and endemic equilibrium points, the condition for local asymptotic stability is $\lambda = \rho - \eta < 0$. For the HIV model, the stability condition for the disease-free equilibrium becomes $\rho - \eta = \varepsilon -\delta < 0$ and for the endemic equilibrium it becomes $\rho - \eta = \delta - \varepsilon < 0$. Therefore, the HIV disease-free equilibrium is locally asymptotically stable if $\varepsilon - \delta < 0$, and the basic reproductive number is then $R_{0} = \frac{\varepsilon }{\delta } < 1$. Similarly, the ELM disease-free equilibrium is locally asymptotically stable if $\beta -r < 0$, and the basic reproductive number is $R_{0} = \frac{\beta }{r} < 1$.

Note that, for zero time delay, the disease-free equilibrium is locally asymptotically stable if $R_{0} < 1$ and unstable if $R_{0} > 1$, and that the endemic equilibrium exists only if $R_{0} > 1$.

### Andronov–Hopf bifurcations of time-delay models

From bifurcation theory [24], Andronov–Hopf (or Hopf) bifurcations exist in an equilibrium solution if the eigenvalues *λ* of the linearized equation about the equilibrium solution satisfy the Andronov–Hopf bifurcation theorem.

#### Theorem 1

(Andronov–Hopf bifurcation theorem)

*A system of time*-*delay ordinary differential equations has an Andronov–Hopf bifucation point if the following conditions are satisfied*:

1. *There exists a critical value of the time delay*$\tau =\tau _{c} > 0$*for which an eigenvalue*$\lambda _{c} = i\phi _{c}$*of the Jacobian of the linearized equations is purely imaginary*, *i*.*e*. $\phi _{c}$*is real and nonzero*.
2. *For*$\tau =\tau _{c}$, *all other eigenvalues have negative real parts*, *and for*$0 < \tau < \tau _{c}$, *all eigenvalues have negative real parts*.
3. *The derivative*$\frac{d (\Re (\lambda ))}{d\tau } \vert _{\tau =\tau _{c}} \neq 0$, *where* ℜ *denotes real part*.

We first look for possible solutions of the characteristic equation (9) for the endemic equilibrium that satisfy condition (C1) by looking for a purely imaginary solution $\lambda _{c} = i\phi _{c}$ with $\phi _{c} > 0$ for $\tau =\tau _{c} >0$.

#### Lemma 1

*Necessary and sufficient conditions for existence of purely imaginary solutions*$\lambda = i\phi $*of the characteristic equation*$\lambda = \rho - \eta e^{-\lambda \tau }$*satisfying condition* (*C*1) *for*$\tau =\tau _{c} > 0$*and*$\phi =\phi _{c} \neq 0$*are* 1) $\eta > 0$*and* 2) $|\rho | < \eta $. *If these conditions are satisfied*, *then the corresponding*$\phi _{c}$*and*$\tau _{c}$*values are given by*

10 $$ \tau _{c} = \frac{1}{\phi _{c}}\cos ^{-1}(\rho /\eta )= \frac{1}{\phi _{c}}\sin ^{-1}(\phi _{c}/\eta ),\quad \phi _{c} = \pm \sqrt{\eta ^{2} - \rho ^{2}}. $$

*Note*: *Since complex solutions must exist in complex conjugate pairs*, *we will assume that*$\phi _{c} = \sqrt{\eta ^{2}-\rho ^{2}} > 0$.

#### Proof

Substituting $\lambda = i\phi $ into (9), separating real and imaginary parts, and solving for *ϕ*, we obtain

11 $$ \rho = \eta \cos (\phi \tau ),\qquad \phi = \eta \sin (\phi \tau ), $$

where $\phi =\sqrt{\eta ^{2} -\rho ^{2}}$ is real and nonzero if and only if $|\rho | < |\eta |$. Then, solving for *τ* in (11), we obtain the expressions for $\tau _{c}$ in (10), and from the second expression for $\tau _{c}$ in (10), we see that a real positive solution for $\tau _{c}$ exists if and only if $\eta > 0$.

The minimum value of $\tau _{c}$ satisfying Eq. (10) also satisfies condition (C1) and therefore it is a possible Andronov–Hopf bifurcation point. □

Then, using the values of *ρ* and *η* for the HIV and ELM delay equations given in Table 3, we find that, as shown in Table 4, possible Andronov–Hopf bifurcations can occur from disease-free equilibrium states for four of the time-delay versions and from endemic equilibrium states for five of the versions. However, we have found that bifurcations from the disease-free states give limit cycles that contain negative population values. For these “limit cycle” regions, the extra condition that the state variable cannot be negative must be added to the mathematical model.

**Table 4:** Andronov–Hopf bifurcation conditions and values for $\tau _{c}$ in HIV and ELM delay models

Version	Disease-free			
	HIV: $R_{0} = \frac{\varepsilon }{\delta }$		ELM: *Λ* = *β* − *r*, $R_{0} = \frac{\beta }{r}$	
	Bifurcation	$\tau _{c}$	Bifurcation	$\tau _{c}$
dnn	$R_{0} < 1$	$\frac{1}{\phi _{c}}\cos ^{-1} (\frac{\varepsilon }{\delta } ) $	$R_{0}<1$	$\frac{1}{\phi _{c}}\cos ^{-1} (\frac{\beta }{r} )$
	*ε*<*δ*	$\phi _{c}= \sqrt{\delta ^{2}-\varepsilon ^{2}}$	*β*<*r*	$\phi _{c} = \sqrt{r^{2}+\beta ^{2}}$
ndn	No	–	No	–
nnd	No	–	No	–
ddn	$R_{0} < 1$	$\frac{\pi }{2\phi _{c}} $	$R_{0}<1$	$\frac{\pi }{2\phi _{c}}$
	*ε*<*δ*	*δ* − *ε*	*β*<*r*	$\phi _{c} = r-\beta $
dnd	$R_{0} < 1$	$\frac{1}{\phi _{c}}\cos ^{-1} (\frac{\varepsilon }{\delta } ) $	$R_{0}<1$	$\frac{1}{\phi _{c}}\cos ^{-1} (\frac{\beta }{r} )$
	*ε*>*δ*	$\phi _{c} = \sqrt{\varepsilon ^{2}-\delta ^{2}}$	*β*<*r*	$\phi _{c} = \sqrt{r^{2}+\beta ^{2}}$
ndd	No	–	No	–
ddd	$R_{0} < 1$	$\frac{\pi }{2\phi _{c}} $	$R_{0}<1$	$\frac{\pi }{2\phi _{c}}$
	*ε*<*δ*	$\phi _{c} = \delta -\varepsilon $	*β*<*r*	$\phi _{c} = r-\beta $

Version	Endemic			
	HIV: $R_{0} = \frac{\varepsilon }{\delta }$		ELM: *Λ* = *β* − *r*, $R_{0} = \frac{\beta }{r}$	
	Bifurcation	$\tau _{c}$	Bifurcation	$\tau _{c}$
dnn	$1 < R_{0} <3$	$\frac{1}{\phi _{c} }\cos ^{-1} (\frac{2\delta -\varepsilon }{\delta } ) $	$1 < R_{0} < 1+\frac{2}{\gamma }$	$\frac{1}{\phi _{c}}\cos ^{-1} (\frac{r-\gamma \varLambda }{r} )$
	*δ*<*ε*<3*δ*	$\phi _{c}= \sqrt{4\delta \varepsilon - \varepsilon ^{2}-3\delta ^{2}}$	0<*γΛ*<2*r*	$\phi _{c} = \sqrt{2r\gamma \varLambda +\gamma ^{2}\varLambda ^{2}}$
ndn	No	–	No	–
nnd	$R_{0} > 1$	$\frac{\pi }{2\phi _{c}} $	$R_{0} > 1$	$\frac{\pi }{2\phi _{c}}$
	*ε*>*δ*	$\phi _{c}= \varepsilon -\delta $	*Λ*>0	$\phi _{c} = \gamma \varLambda $
ddn	No	–	No	–
dnd	$R_{0} > 1$	$\frac{1}{\phi _{c}}\cos ^{-1} (\frac{\delta }{\varepsilon } ) $	$R_{0} > 1$	$\frac{1}{\phi _{c}}\cos ^{-1} (\frac{r}{r+\gamma \varLambda } )$
	*ε*>*δ*	$\phi _{c} = \sqrt{\varepsilon ^{2}-\delta ^{2}}$	*Λ*>0	$\phi _{c} = \sqrt{\gamma ^{2}\varLambda ^{2}+2r\gamma \varLambda }$
ndd	$R_{0} > 3$	$\frac{1}{\phi _{c}}\cos ^{-1} (\frac{\delta }{\varepsilon -2\delta } ) $	$R_{0} > 1 +\frac{2}{\gamma } $	$\frac{1}{\phi _{c}}\cos ^{-1} (\frac{r}{\gamma \varLambda -r} )$
	*ε*>3*δ*	$\phi _{c}= \sqrt{\varepsilon ^{2}+3\delta ^{2}-4\delta \varepsilon } $	*γΛ*>2*r*	$\phi _{c} = \sqrt{\gamma ^{2}\varLambda ^{2}-2r\gamma \varLambda }$
ddd	$R_{0}>1$	$\frac{\pi }{2\phi _{c}} $	$R_{0} > 1$	$\frac{\pi }{2\phi _{c}}$
	*ε*>*δ*	$\phi _{c} = \varepsilon -\delta $	*Λ*>0	$\phi _{c} = \gamma \varLambda $

In the remainder of this paper, we will only consider bifurcations from endemic equilibrium states.

#### Lemma 2

*If the necessary and sufficient conditions stated in Lemma *1*are satisfied for an endemic equilibrium state*, *then the critical*$\tau _{c} > 0$*defined in Lemma *1*also satisfies conditions* (C2) *and* (C3) *of the Andronov–Hopf bifurcation theorem*.

#### Proof

Let $\lambda = \mu + i\phi $, where *μ* and *ϕ* are real, and we can assume that $\phi \geq 0$.

**Figure 1:**
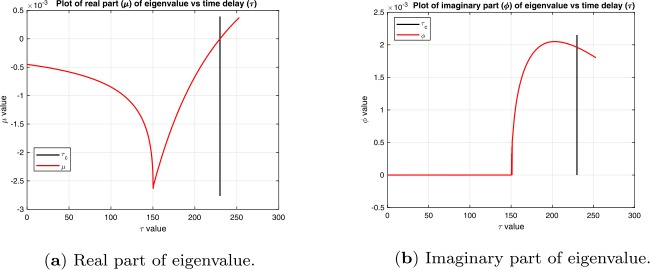
Plots of real and imaginary parts of eigenvalues vs time delay *τ* for delay differential equation. The vertical black line is at $\tau =\tau _{c}$

Note: An example of the variation of *μ* and *ϕ* as *τ* is increased that is used in this lemma to prove condition (C2) is shown in Fig. 1. The real and imaginary parts of (9) are $\mu = \rho -\eta e^{-\mu \tau }\cos (\phi \tau )$, $\phi = \eta e^{-\mu \tau }\sin (\phi \tau )$, and the real and imaginary parts of the derivative of the characteristic equation (9) with respect to *τ* can be written as:

12 $$\begin{aligned}& \frac{d\mu }{d\tau } = \frac{1}{\Delta }\eta e^{-\mu \tau } \biggl( \mu \bigl(1-\tau \eta e^{-\mu \tau }\bigr) -2 \mu \sin ^{2}\biggl( \frac{\phi \tau }{2}\biggr)+\phi \sin (\phi \tau ) \biggr) , \end{aligned}$$

13 $$\begin{aligned}& \frac{d\phi }{d\tau } = \frac{1}{\Delta }\eta e^{-\mu \tau } \biggl( \phi \bigl(1-\tau \eta e^{-\mu \tau }\bigr) -2\phi \sin ^{2}\biggl( \frac{\phi \tau }{2}\biggr)-\mu \sin (\phi \tau ) \biggr), \end{aligned}$$

where

14 $$ \Delta = \bigl\vert {1-\eta \tau e^{-\lambda \tau }} \bigr\vert ^{2} = \bigl(1- \tau \eta e^{-\mu \tau }\bigr)^{2}+4 \tau \eta e^{-\mu \tau }\sin ^{2}\biggl( \frac{\phi \tau }{2} \biggr). $$

Clearly, *μ* and *ϕ* are continuous functions of *τ*, but the derivatives can be discontinuous if $\Delta = 0$.

We consider three cases:

1. $1-\tau \eta e^{-\mu \tau } >0$. At $\tau =0$, we have $\mu = \rho - \eta < 0$ and $\phi = 0$. Further, since $\mu < 0$ and $\Delta =1 > 0$, we have $\frac{d\mu }{d\tau } < 0$ and $\frac{d\phi }{d\tau } = 0$ at $\tau = 0$. As *τ* increases, *μ* will decrease and remain negative and *ϕ* will remain zero. Therefore, the eigenvalue will be real and negative.
2. $1-\tau \eta e^{-\mu \tau } =0$. Since $\mu < 0$, this condition must occur at some point $\tau =\tau _{d} > 0$. Also, since $\phi = 0$ for $\tau < \tau _{d}$, $\Delta = 0$ at $\tau = \tau _{d}$. The derivatives $\frac{d\mu }{d\tau }$ and $\frac{d\phi }{d\tau }$ will therefore be discontinuous at $\tau _{d}$. Then, since $\phi = 0$ is actually an unstable solution of (13), the discontinuity at $\tau _{d}$ will make *ϕ* become nonzero.
3. $1-\tau \eta e^{-\mu \tau } < 0$. As *τ* increases, *ϕ* becomes non-negative. If $\phi > 0$, then, for *τ* in the positive neighborhood of $\tau _{d}$, we have $\mu < \rho - \eta < 0$, and therefore for small *ϕ*, $\frac{d\phi }{d\tau } > 0$ if $\phi > 0$ and $\frac{d\phi }{d\tau } < 0$ if $\phi < 0$. Figure 1 shows the case of $\phi > 0$.
  In both cases, $\mu < 0$ and therefore $\frac{d\mu }{d\tau } > 0$ and *μ* increases monotonically until $\mu = 0$. At this point, we see that the Andronov–Hopf bifurcation condition (C1) will be satisfied and we can identify the *τ* value as the critical value $\tau _{c}$ and the *ϕ* value as the critical value $\phi _{c}$ defined in Lemma 1. Since $\mu < 0$ for $\tau < \tau _{c}$, we have proved condition (C2) for $\tau _{c}$.

Finally, substituting $\mu = 0$, $\phi =\phi _{c}$ and $\tau =\tau _{c}$ into (12) and (14), we have

15 $$ \frac{d\mu }{d\tau }\bigg|_{\tau =\tau _{c}} = \frac{\phi _{c}^{2}}{(1-\tau _{c} \eta )^{2} + 2\tau _{c}(\eta -\rho )}. $$

Since $\eta - \rho > 0$, the denominator is always positive and therefore condition (C3) is satisfied. Also, since $\frac{d\mu }{d\tau } \vert _{\tau =\tau _{c}}>0$, the bifurcation will occur as *τ* increases to $\tau _{c}$. The proof is complete. □

Combining the results of Lemmas 1 and 2, we obtain the following theorem.

#### Theorem 2

*Necessary and sufficient conditions for the existence of an Andronov–Hopf bifurcation in the endemic equilibrium points in equation* (8) *are*$\eta > 0$*and*$|\rho | < \eta $. *Then the Andronov–Hopf bifurcation exists at a critical time delay*:

16 $$ \tau _{c} = \frac{1}{\phi _{c}}\cos ^{-1}(\rho /\eta ) = \frac{1}{\phi _{c}}\sin ^{-1}(\phi _{c}/\eta ), $$

*where*$\phi _{c} = \sqrt{\eta ^{2} - \rho ^{2}}$. *Further*, *the bifurcation occurs asτincreases through*$\tau _{c}$.

## Equilibrium points, stability and Neimark–Sacker bifurcations of difference equation models

### Equilibrium points and basic reproduction numbers

The equilibrium points $w^{*}$ for the difference equation models are obtained by setting $w_{n+1} = w_{n} = w_{n-m}=w^{*}$ in equation (6). For each model, we obtain a trivial equilibrium point $w^{*} = 0$ and an endemic equilibrium point given by

17 $$ \mbox{HIV:}\quad w^{*}= 0,\qquad w^{*} = 1 - \frac{\delta }{\varepsilon };\qquad \mbox{ELM:}\quad w^{*} = 0,\qquad w^{*} = K \biggl(1-\frac{r}{\beta } \biggr)^{ \frac{1}{\gamma }}. $$

For the HIV model, the endemic equilibrium exists only if $\varepsilon > \delta $, and for the extended logistic model, the endemic equilibrium exists only if $\beta > r$. The equilibrium points in (17) are also equilibrium points for the differential equation models.

To obtain the basic reproductive numbers $R_{0}$ for local stability of the disease-free equilibrium points, we check the eigenvalues of the linearized system at the disease-free equilibrium. We let $y_{n} = w_{n}-w^{*}$ and assume that $y_{n}$ is small. Then the linearized equations are:

18 $$ \mbox{HIV:}\quad y_{n+1} = \bigl(1-h_{m}( \delta - \varepsilon )\bigr)y_{n},\qquad \mbox{ELM:}\quad y_{n+1} = \bigl(1-h_{m}(r-\beta )\bigr)y_{n}. $$

Therefore, the HIV disease-free equilibrium is locally asymptotically stable if $|1-h_{m}(\delta - \varepsilon )| < 1$, i.e., if $R_{0} = \frac{\varepsilon }{\delta } < 1$. Similarly, the ELM disease-free equilibrium is locally asymptotically stable if $R_{0} = |1-h_{m}(r-\beta )| < 1$, i.e., if $R_{0} = \frac{\beta }{r} < 1$.

Note that in both cases, the disease-free equilibrium is locally asymptotically stable if $R_{0} < 1$, whereas the endemic equilibrium exists only if $R_{0} > 1$. Also, as noted in section (4), the stability conditions for the numerical stability of the forward Euler approximation put an upper limit on the step size $h=h_{m}\tau $ in the difference equations (18).

### Conditions for Neimark–Sacker bifurcations

We obtain the conditions for Neimark–Sacker bifurcations of the endemic equilibrium solutions of the nonlinear HIV and ELM equations (18) from the linearized equations about the endemic equilibrium points.

To obtain the linearized, time-delayed versions of the nonlinear difference equations, we define $y_{n+1} = w_{n+1} - w^{*}$, $y_{n} = w_{n} - w^{*}$ and $y_{n-m} = w_{n-m} - w^{*}$. Then we obtain

19 $$ y_{n+1} = y_{n} + \rho h_{m}\tau y_{n} - \eta h_{m}\tau y_{n-m}, $$

where *ρ* and *η* are constants and $h_{m}=1/m$. The values of these constants for the seven linearized versions of the discrete HIV and ELM equations are the same as the values given in Table 3 for the seven linearized versions of the HIV and ELM differential equations in Table 1.

Then, assuming a trial solution of the form $y_{n} = \lambda ^{n}$ for (19), we obtain the characteristic equation, which it is convenient to write in the alternative forms

20 $$\begin{aligned}& P_{1}(\lambda ) = \lambda ^{m}(\lambda -1) - h_{m}\tau \bigl(\rho \lambda ^{m} -\eta \bigr) =0, \end{aligned}$$

21 $$\begin{aligned}& P_{2}(\lambda ) = \lambda -1 - h_{m}\tau \bigl(\rho - \eta \lambda ^{-m}\bigr) = 0, \end{aligned}$$

22 $$\begin{aligned}& P_{3}(\lambda ) = \lambda ^{\frac{1}{2}}-\lambda ^{-\frac{1}{2}} -h_{m} \tau \bigl(\rho \lambda ^{-\frac{1}{2}} - \eta \lambda ^{-(m+\frac{1}{2})}\bigr) =0. \end{aligned}$$

From the real and imaginary parts of the characteristic equations in (20)–(22), we obtain the conditions

23 $$\begin{aligned}& m r^{m+1}\cos \bigl((m+1)\omega \bigr) = (m+\rho \tau )r^{m} \cos (m\omega ) + \eta \tau , \end{aligned}$$

24 $$\begin{aligned}& m r^{m+1}\sin \bigl((m+1)\omega \bigr) = (m+\rho \tau )r^{m} \sin (m\omega ), \end{aligned}$$

25 $$\begin{aligned}& m r^{m+1}\cos (\omega ) = (m+\rho \tau )r^{m} +\eta \tau \cos (m \omega ), \end{aligned}$$

26 $$\begin{aligned}& m r^{m+1}\sin (\omega ) = \eta \tau \sin (m\omega ), \end{aligned}$$

27 $$\begin{aligned}& m r^{m+1}\cos \biggl(\frac{1}{2}\omega \biggr) = (m+\rho \tau ) r^{m} \cos \biggl( \frac{1}{2}\omega \biggr)-\eta \tau \cos \biggl(\biggl(m+\frac{1}{2}\biggr)\omega \biggr), \end{aligned}$$

28 $$\begin{aligned}& m r^{m+1} \sin \biggl(\frac{1}{2}\omega \biggr) = - (m+ \rho \tau ) r^{m} \sin \biggl( \frac{1}{2}\omega \biggr)- \eta \tau \sin \biggl(\biggl(m+\frac{1}{2}\biggr) \omega \biggr). \end{aligned}$$

From bifurcation theory [24], the conditions for the existence of a Neimark–Sacker bifurcation are as follows.

#### Theorem 3

(Neimark–Sacker bifurcation theorem)

*A Neimark–Sacker bifurcation point occurs if there exists a time delay*$\tau =\tau _{c}$*such that the eigenvalues*$\lambda (\tau ) = r(\tau )e^{i\omega (\tau )}$*of a linearized system of nonlinear difference equations satisfy the following conditions*:

1. *For*$\tau = \tau _{c}$, *there exists a complex conjugate pair of eigenvalues on the unit circle*, *i*.*e*., $r(\tau _{c}) = 1$, $\lambda (\tau _{c}) = e^{\pm i\omega _{c}}$*and*$0 < \omega (\tau _{c}) = \omega _{c} < \pi $.
2. *At*$\tau = \tau _{c}$, *all other eigenvalues are inside the unit circle and*, *for*$0< \tau < \tau _{c}$, *all eigenvalues are inside the unit circle*.
3. $r'(\tau _{c}) = \frac{d r(\tau )}{d\tau } \vert _{ \tau =\tau _{c}}\neq 0$.
4. $e^{ik\omega _{c}}\neq 1$, *for*$k=1,2,3,4$.
5. $\Re [e^{-i\omega _{c}}c_{1} (\tau _{c} ) ]\neq 0$, *where*$c_{1}(\tau _{c})$*is a critical function that determines the direction and stability of Neimark–Sacker bifurcations* (*see*, *e*.*g*., [19, 24] *and the section on direction and stability of the Neimark–Sacker bifurcations*, *Sect*. 7).

*If the condition* (C5) *of the Neimark–Sacker theorem is satisfied*, *then an invariant closed curve*, *topologically equivalent to a circle*, *will occur forτin a one sided neighborhood of*$\tau _{c}$. *The radius of the invariant curve will grow like*$O (\sqrt{ |\tau -\tau _{c} |} )$. *One of the four cases below applies*:

1. $r' (\tau _{c} )>0$. *The endemic equilibrium point is locally asymptotically stable for*$\tau <\tau _{c}$*and unstable for*$\tau >\tau _{c}$.
  - $\Re [e^{-i\omega _{c}}c_{1} (\tau _{c} ) ]<0$. *An attracting invariant closed curve exists for*$\tau >\tau _{c}$.
  - $\Re [e^{-i\omega _{c}}c_{1} (\tau _{c} ) ] >0$. *A repelling invariant closed curve exists for*$\tau >\tau _{c}$.
2. $r' (\tau _{c} )<0$. *The endemic equilibrium point is unstable for*$\tau < \tau _{c}$*and locally asymptotically stable for*$\tau >\tau _{c}$.
  - $\Re [e^{-i\omega _{c}}c_{1} (\tau _{c} ) ]<0$. *An attracting invariant closed curve exists for*$\tau <\tau _{c}$.
  - $\Re [e^{-i\omega _{c}}c_{1} (\tau _{c} ) ]>0$. *A repelling invariant closed curve exists for*$\tau <\tau _{c}$.

We first look for solutions of the characteristic equations (20)–(22) that satisfy condition (C1) of Theorem 3.

#### Lemma 3

*A complex conjugate pair of eigenvalues of the characteristic equations* (20)*–*(22) *lie on the unit circle with*$\lambda = e^{i\omega _{c}}$*for a positive value of the delayτif the following conditions are satisfied*:

1. *A positive real solution*$\omega =\omega _{c}$, $0< \omega _{c} < \pi $, *exists for the equation*
  29 $$ R(\omega )=\rho \cos \biggl(\frac{1}{2}\omega \biggr) - \eta \cos \biggl(\biggl(m+\frac{1}{2}\biggr) \omega \biggr) = 0. $$
2. *A positive value of*$\tau = \tau _{c}$*exists such that*$\tau _{c}= \tau _{1}(\omega _{c})= \tau _{2}(\omega _{c})=\tau _{3}( \omega _{c})$, *where*
  30 $$ \begin{aligned} &\tau _{1}(\omega )=\frac{m}{\rho } \biggl( \frac{\sin ((m+1)\omega )}{\sin (m\omega )} -1 \biggr)= \frac{2m\cos ((m+\frac{1}{2})\omega )\sin (\frac{1}{2}\omega )}{\rho \sin (m\omega )} , \\ &\tau _{2}(\omega )= \frac{2m\sin ^{2}(\frac{1}{2}\omega )}{\eta \cos (m\omega )-\rho },\qquad \tau _{3}(\omega )= \frac{m\sin (\omega )}{\eta \sin (m\omega )}. \end{aligned} $$

*Note*: *Since any complex eigenvalues must occur in complex conjugate pairs*, *existence of an eigenvalue with*$\omega =\omega _{c} > 0$*implies existence of an eigenvalue with*$\omega =-\omega _{c} < 0$.

#### Proof

Taking the real part of the characteristic equation $P_{3}(\lambda )=0$ in (22) for $\lambda = e^{\pm i\omega }$, we obtain the condition in (29). Then, taking the imaginary part of $P_{1}(\lambda ) = 0$, and the real and imaginary parts of $P_{2}(\lambda ) =0$, for $\lambda = e^{\pm i\omega }$, we obtain the formulas for $\tau _{1}(\omega )$, $\tau _{2}(\omega )$, $\tau _{3}(\omega )$ in (30). Therefore, if conditions 1 and 2 of the lemma are satisfied, then $\lambda _{c} = e^{i\omega _{c}}$ is a solution of the characteristic equations in (20)–(22) for a real positive value of the time delay. □

We now prove the following lemma.

#### Lemma 4

*Necessary and sufficient conditions for the critical value*$\tau _{c} > 0$*defined in Lemma *3*to exist are*: $|\rho | < \eta $*and*$\eta > 0$.

#### Proof

If $\lambda = r$ for real *r*, then the characteristic equation $P_{2}(\lambda )=0$ in (21) is $m(r-1) = \tau (\rho - \eta r^{-m})$. Then, differentiating this equation with respect to *τ*, we have

31 $$ \frac{dr}{d\tau } = \frac{\rho -\eta }{m(1-\eta \tau )}. $$

For $\tau = 0$, a real solution of the characteristic equation $m(r-1) = \tau (\rho - \eta r^{-m})$ is $r=1$ and, at $r=1$, the derivative $\frac{dr}{d \tau } = \frac{\rho -\eta }{m}$. Therefore, if $\rho -\eta \geq 0$, then a real eigenvalue will remain on or move outside the unit circle for $\tau > 0$, and hence $\tau _{c}$ cannot be a Neimark–Sacker bifurcation point. Therefore, $\rho -\eta < 0$ is a necessary condition for real eigenvalues to lie inside the unit circle in the positive neighborhood of $\tau =0$.

We next prove that necessary and sufficient conditions for $\tau _{c} > 0$ to exist are that $\eta ^{2} - \rho ^{2} > 0$. From the formulas for $\tau _{2}(\omega _{c})$ and $\tau _{3}(\omega _{c})$ in (30) and the condition that $0 < \omega _{c} < \pi $, it can be seen that necessary conditions for $\tau _{2}(\omega _{c})$ and $\tau _{3}(\omega _{c})$ to be positive are that $\sin (\omega _{c}) > 0$ and $\sin (\frac{1}{2}\omega _{c}) > 0$. Therefore, necessary conditions for $\tau _{c} = \tau _{2}(\omega _{c}) = \tau _{3}(\omega _{c})> 0$ to exist are

32 $$ \eta \cos (m\omega _{c}) > \rho , \qquad \eta \sin (m \omega _{c}) > 0,\quad \mbox{and therefore} \quad \eta ^{2} > \rho ^{2}. $$

Next, if $\rho -\eta < 0$ and $\eta ^{2} - \rho ^{2} > 0$, then $\rho + \eta > 0$. Then, since $\rho - \eta <0$ and $\rho +\eta > 0$, it is necessary that $\eta > 0$ and $|\rho | < \eta $.

We now prove that the conditions are sufficient. In Eq. (29), the function $R(\omega )$ is a continuous function of *ω* for $0 \leq \omega \leq \pi $. We have $R(0) = \rho - \eta < 0$ and $R(\frac{2\pi }{2m+1}) = \rho \cos (\frac{\pi }{2m+1}) + \eta > 0$. Therefore, from the intermediate value theorem of elementary calculus, $R(\omega ) = 0$ for at least one $\omega \in (0,\frac{2\pi }{2m+1})$.

Therefore, from Lemma 3, $\tau = \tau _{c}$ exists and satisfies condition (C1) of the Neimark–Sacker theorem. The proof is complete □

We now prove condition (C2).

#### Lemma 5

*If the necessary and sufficient conditions stated in Lemma *4*are satisfied*, *then the critical*$\tau _{c} > 0$*defined in Lemma *3*also satisfies condition* (C2) *of the Neimark–Sacker bifurcation theorem*.

**Figure 2:**
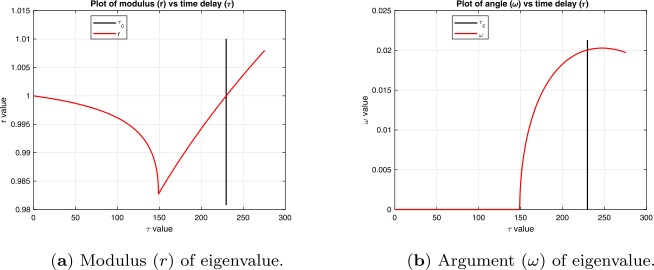
Plots of moduli and arguments of eigenvalues vs time delay *τ*. The vertical black line is at $\tau =\tau _{c}$

Note: An example of the variation of *r* and *ω* as *τ* is increased that is used in this lemma to prove condition (C2) is shown in Fig. 2.

#### Proof

Let $\lambda = r e^{i \omega }$, where $r \geq 0$ and $\omega \neq 0$ are real. Also, since any complex eigenvalues must occur in complex conjugate pairs, we can assume that $0 < \omega < \pi $.

Then, differentiating Eqs. (23) and (24) with respect to *τ* and simplifying using (23)–(26), we can write the derivatives for *r* and *ω* in the forms

33 $$\begin{aligned}& \begin{aligned}[b] \frac{dr}{d\tau } &= \frac{1}{\Delta }\bigl( \bigl(r^{m+1}-\eta \tau \bigr) \bigl( \rho r^{m} - \eta \bigr)\cos (\omega ) + \rho \eta \tau r^{m} \bigl(\cos ( \omega )-\cos (m \omega )\bigr) \\ &\quad {} + \eta r^{m+1} \bigl(\cos (\omega )-\cos \bigl((m+1)\omega \bigr) \bigr) + \eta ^{2} \tau \bigl(1-\cos (\omega ) \bigr)\bigr) \\ &= \frac{1}{\tau \Delta } \biggl(\bigl(r^{m+1}-\eta \tau \bigr) \bigl(\rho r^{m}-\eta \bigr) +2 \eta \tau r^{m} \sin ^{2}\biggl(\frac{1}{2} m\omega \biggr) \\ &\quad {} + 2\bigl(\eta -\rho r^{m}\bigr)r^{m+1} \biggl(\sin ^{2}\biggl(\frac{1}{2}\bigl((m+1) \omega \bigr)\biggr)+\sin ^{2}\biggl(\frac{1}{2}\omega \biggr) \biggr) \biggr), \end{aligned} \end{aligned}$$

34 $$\begin{aligned}& \frac{d\omega }{d\tau }=\frac{r^{m} (1-\rho \tau )\eta \sin (m\omega )}{\Delta }, \end{aligned}$$

where

35 $$ \Delta = m \biggl(\bigl(r^{m+1}-\eta \tau \bigr)^{2}+4 r^{m+1} \eta \tau \sin ^{2}\biggl( \frac{(m+1)\omega }{2} \biggr) \biggr). $$

As for the Andronov–Hopf bifurcation, we consider three cases.

1. $r^{m+1} - \eta \tau > 0$. Then, at $\tau = 0$, we have from (20) $\lambda = r= 1$, and $\omega = 0$, and from (34) that $\frac{d\omega }{d\tau } = 0$. Hence, in the positive neighborhood of $\tau = 0$, we have $\omega = 0$ and then
  36 $$ \Delta = m\bigl(r^{m+1} - \eta \tau \bigr)^{2} \quad \mbox{and}\quad \frac{dr}{d\tau } = \frac{\rho r^{m} - \eta }{m(r^{m+1}-\eta \tau )} < 0. $$
  Therefore, as shown in Fig. 2, for $r^{m+1} - \eta \tau > 0$, the modulus *r* decreases and the angle *ω* remains zero.
2. $r^{m+1} - \eta \tau = 0$. At $\tau = \tau _{d} = \frac{r^{m+1}}{\eta }$, the denominator $\Delta =0$ and, as shown in Fig. 2, the derivatives $\frac{dr}{d\tau }$ and $\frac{d\omega }{d\tau }$ are discontinuous. Then, since $\omega =0$ is an unstable solution of (34), *ω* becomes nonzero (positive in Fig. 2) in the positive neighborhood of $\tau _{d}$.
3. $r^{m+1} - \eta \tau < 0$. In this case, it can be seen from Eq. (33) that $\frac{dr}{d\tau } > 0$, and therefore *r* increases.
  We now show that $r^{m+1}-\eta \tau < 0$ for all $\tau \leq \tau _{c}$. From (26), we have
  37 $$ \frac{r^{m+1}}{\eta \tau } = \frac{\sin (m\omega )}{m\sin (\omega )}. $$
  Also, from Lemma 4, we have the condition that, for $\omega \neq 0$, the critical value of $\omega _{c} \in (0,\frac{2\pi }{2m+1})$. It is straightforward to prove that
  38 $$ \frac{\sin (m\omega )}{m\sin (\omega )} < 1\quad \mbox{for } \omega \in \biggl(0,\frac{2\pi }{2m+1} \biggr]. $$
  Therefore $r^{m+1}-\eta \tau < 0$ for $\tau _{d} < \tau \leq \tau _{c}$, and hence *r* is a continuous, monotonically increasing function of *τ* for $\tau _{d} \leq \tau \leq \tau _{c}$. Therefore $\tau = \tau _{c}$ is the minimum value of *τ* for which $r=1$. The proof is complete.

 □

We now prove condition (C3).

#### Lemma 6

*If the necessary and sufficient conditions stated in Lemma *4*are satisfied*, *then the critical*$\tau _{c} > 0$*defined in Lemma *3*also satisfies condition* (C3) *of the Neimark–Sacker bifurcation theorem*.

#### Proof

As shown in Lemma 5, $\frac{dr}{d\tau } > 0$ for $\tau _{d} < \tau \leq \tau _{c}$, and therefore condition (C3) is satisfied. □

We now prove condition (C4).

#### Lemma 7

*If the necessary and sufficient conditions given in Lemma *4*for*$\tau _{c} > 0$*to exist are satisfied and if*$m > 1$, *then the corresponding critical argument*$\omega = \omega _{c}$*satisfies the conditions*$e^{ik\omega _{c}}\neq 1$*for*$k=1,2,3,4$.

#### Proof

Since $0 < \omega _{c} < \frac{2\pi }{2m+1}$ and $m >1$, we have $0 < k\omega _{c} < 2\pi $ for $k=1,2,3,4$ and therefore $e^{ik\omega _{c}}\neq 1$. □

Then, combining Lemmas 3–7, we have the following theorem.

#### Theorem 4

*If the necessary and sufficient conditions stated in Lemma *4*are satisfied and if*$m>1$, *then a critical time delay*$\tau _{c} > 0$*exists for Eq*. (19) *that satisfies conditions* (C1), (C2), (C3) *and* (C4) *of the Neimark–Sacker bifurcation theorem*.

Then, from Theorem 4, we have shown that the conditions for possible Neimark–Sacker bifurcations for the seven versions of the discrete HIV and extended logistic model equations are the same as the values given in Table 4 for the seven versions of the HIV and ELM differential equations in Table 1.

## Direction and stability of the Neimark–Sacker bifurcations

In this section, we find the direction and stability of Neimark–Sacker bifurcations by studying condition (C5) of the Neimark–Sacker theorem for the five versions $(\mathrm{dnn},\mathrm{nnd}, \mathrm{dnd}, \mathrm{ndd},\mathrm{ddd})$ of the time-delay difference equations that show bifurcations. To prove condition (C5), we follow the method given in Li [19] and Kuznetsov [24] to compute the function called $c_{1}(\tau )$ for the critical value $\tau = \tau _{c}$. The main ideas in the derivation are to replace the single nonlinear difference equations of order $m+1$ in the perturbations $y_{n} = w_{n} -w^{*}$ by a system of $m+1$ first-order difference equations and then to examine the terms up to third order in $y_{n}$ in the Taylor series expansions of the right-hand sides of these systems of first-order nonlinear difference equations. The critical function $c_{1}(\tau _{c})$ can then be computed from these terms.

The terms up to third order of the nonlinear difference equations for the five versions of the HIV and ELM models are shown in Table 5.

**Table 5:** Nonlinear discrete HIV and ELM equations for versions with Neimark–Sacker bifurcations (values of *ρ* and *η* are given in Table 3)

Version	HIV $\psi = h_{m}\tau $
dnn	$y_{n+1}=y_{n}+\varepsilon \psi y_{n}-\delta \psi y_{n-m} -\varepsilon \psi (y_{n}+w^{*})^{2}$
second order	$y_{n+1} = y_{n} + \rho \psi y_{n}-\eta \psi y_{n-m}-2\varepsilon \psi y^{2}_{n} $
nnd	$y_{n+1}=y_{n}-\delta \psi y_{n}+\varepsilon \psi y_{n-m} -\varepsilon \psi (y_{n}+w^{*})(y_{n-m}+w^{*})$
second order	$y_{n+1} = y_{n}- \eta \psi y_{n-m} -\varepsilon \psi (y_{n}y_{n-m}+y_{n-m}y_{n})$
dnd	$y_{n+1}=y_{n}-\varepsilon \psi y_{n}-\delta \psi y_{n-m} -\varepsilon \psi (y_{n}+w^{*})(y_{n-m}+w^{*})$
second order	$y_{n+1}=y_{n} +\rho \psi y_{n}-\eta \psi y_{n-m}-\varepsilon \psi (y_{n}y_{n-m}+y_{n-m}y_{n})$
ndd	$y_{n+1}=y_{n}-\delta \psi y_{n}+\varepsilon \psi y_{n-m} -\varepsilon \psi (y_{n-m}+w^{*})^{2}$
second order	$y_{n+1}=y_{n} +\rho \psi y_{n}-\eta \psi y_{n-m} -2 \varepsilon \psi y_{n-m}^{2}$
ddd	$y_{n+1}=y_{n}+(\varepsilon -\delta ) \psi y_{n-m} -\varepsilon \psi (y_{n-m}+w^{*})^{2}$
second order	$y_{n+1} = y_{n}-\eta \psi y_{n-m} -2\varepsilon \psi y^{2}_{n-m} $

Version	ELM $\psi = h_{m}\tau $, *Λ* = *β* − *r*, $\nu =\frac{\beta }{K}(\frac{\varLambda }{\beta })^{\frac{\gamma -1}{\gamma }}$, $\xi =\frac{\beta }{K^{2}}(\frac{\varLambda }{\beta })^{\frac{\gamma -2}{\gamma }}$
dnn	$y_{n+1} = y_{n} +\beta \psi y_{n}-r\psi y_{n-m}-\alpha \psi \frac{\beta }{K^{\gamma }}(y_{n}+w^{*})^{\gamma +1}$
third order	$y_{n+1} = y_{n} +\rho \psi y_{n}-\eta \psi y_{n-m}- (\gamma ^{2}+\gamma )\nu \psi y_{n}^{2}- (\gamma ^{3}-\gamma )\xi \psi y_{n}^{3}$
nnd	$y_{n+1} = y_{n} +\psi \varLambda y_{n}-\alpha \psi \frac{\beta }{K^{\gamma }} (y_{n}+w^{*})(y_{n-m}+w^{*})^{\gamma }$
third order	$\begin{array}[t]{l} y_{n+1} = y_{n} -\eta \psi y_{n-m} -\nu \psi [(\gamma ^{2}-\gamma ) y_{n-m}^{2}+\gamma ( y_{n} y_{n-m}+y_{n-m} y_{n})] \\ \hphantom{y_{n+1} ={}}{}-\xi \psi [(\gamma ^{3}-3\gamma ^{2}+2\gamma )y_{n-m}^{3} \\ \hphantom{y_{n+1} ={}}{}+(\gamma ^{2}-\gamma )(y_{n} y_{n-m}^{2}+ y_{n-m} y_{n}y_{n-m}+ y_{n-m}^{2} y_{n})]\end{array}$
dnd	$\begin{array}[t]{l} y_{n+1} = y_{n} +\alpha \psi (1+\beta )y_{n}-r\psi y_{n-m} \\ \hphantom{y_{n+1} ={}}{}-\alpha \psi \frac{\beta }{K^{\gamma }}(y_{n}+w^{*})(y_{n-m}+w^{*})^{\gamma }\end{array}$
third order	$\begin{array}[t]{l} y_{n+1} = y_{n} + \rho \psi y_{n} -\eta \psi y_{n-m} \\ \hphantom{y_{n+1} ={}}{}-\nu \psi [(\gamma ^{2}-\gamma ) y_{n-m}^{2}+\gamma ( y_{n} y_{n-m}+y_{n-m} y_{n})] \\ \hphantom{y_{n+1} ={}}{}-\xi \psi [(\gamma ^{3}-3\gamma ^{2}+2\gamma )y_{n-m}^{3} \\ \hphantom{y_{n+1} ={}}{}+(\gamma ^{2}-\gamma )(y_{n} y_{n-m}^{2}+ y_{n-m} y_{n}y_{n-m}+ y_{n-m}^{2} y_{n})]\end{array}$
ndd	$y_{n+1} = y_{n} -r\psi y_{n}+\alpha \psi (1+\beta )y_{n-m}-\alpha \psi \frac{\beta }{K^{\gamma }}(y_{n-m}+w^{*})^{\gamma +1}$
third order	$\begin{array}[t]{l}y_{n+1} = y_{n} +\rho \psi y_{n}-\eta \psi y_{n-m} -(\gamma ^{2}+\gamma )\nu \psi y_{n-m}^{2} \\ \hphantom{y_{n+1} ={}}{}- (\gamma ^{3}-\gamma )\xi \psi y_{n-m}^{3}\end{array}$
ddd	$y_{n+1} = y_{n} +\psi \varLambda y_{n-m}-\alpha \psi \frac{\beta }{K^{\gamma }}(y_{n-m}+w^{*})^{\gamma +1}$
third order	$y_{n+1} = y_{n} -\eta \psi y_{n-m}-(\gamma ^{2}+\gamma )\nu \psi y_{n-m}^{2}- (\gamma ^{3}-\gamma )\xi \psi y_{n-m}^{3}$

We now introduce a vector $Y_{n} = (y_{n},y_{n-1},y_{n-2},\ldots ,y_{n-m})^{T}$, $n \geq m$ and write the system of first-order difference equations up to third order in the Taylor series expansion in the form

39 $$ Y_{n+1} = \mathcal{A} Y_{n} + \frac{1}{2}\mathcal{B}(Y_{n},Y_{n}) + \frac{1}{6}\mathcal{C}(Y_{n},Y_{n},Y_{n})+O \bigl( \Vert Y_{n} \Vert ^{4}\bigr). $$

In (39), $\mathcal{A}$ is the Jacobian of the system of $m+1$ first-order difference equations and $\mathcal{B}$ and $\mathcal{C}$ are the second- and third-order terms in the system. We will first discuss the Jacobian and eigenvectors and then give the formulas for the second- and third-order terms. Finally, we will derive the formula for the critical coefficient $c_{1}(\tau _{c})$ for the five HIV and ELM versions that have Neimark–Sacker bifurcations.

### The Jacobian matrix and eigenvectors

The Jacobian matrix $\mathcal{A}= J(\tau )$ for the linearized system of $m+1$ first-order equations corresponding to the nonlinear difference equations in (19) is given by

40 $$ J(\tau ) = \begin{pmatrix} 1+\rho h_{m}\tau & 0& \cdots & 0& -\eta h_{m}\tau \\ 1& 0& \cdots & 0& 0 \\ 0& 1& \cdots & 0& 0 \\ \vdots & \vdots & \ddots & \vdots & \vdots \\ 0& 0& \cdots & 1& 0 \end{pmatrix} . $$

Since the Jacobian matrix is the companion matrix [43] of the characteristic equation (20), the eigenvalues of the Jacobian matrix and the characteristic equation are the same. For a critical value $\tau _{c}$, a solution of the characteristic equation (20) is $\lambda _{c} = e^{i\omega _{c}}$. Then the eigenvector of $J(\tau )$ for the eigenvalue $\lambda _{c} = e^{i\omega _{c}}$ can be written in the form

41 $$ q \bigl(e^{i\omega _{c}} \bigr)= \bigl(e^{im\omega _{c}},e^{i (m-1 )\omega _{c}}, \ldots ,e^{i\omega _{c}},1 \bigr)^{T}, $$

where we have chosen $q_{m} =1$ as a normalization constant.

Following Li [19], we also introduce the adjoint eigenvector

42 $$ r \bigl(e^{i\omega _{c}} \bigr)=D \bigl(1,\sigma e^{im\omega _{c}}, \sigma e^{i (m-1 )\omega _{c}},\ldots ,\sigma e^{i2\omega _{c}}, \sigma e^{i\omega _{c}} \bigr)^{T}, $$

of the transposed Jacobian matrix $J(\tau _{c})^{T}$. In (42), $\sigma =-\eta h_{m}\tau _{c}$, and $D= (e^{-im\omega _{c}}+m\sigma e^{i\omega _{c}} )^{-1}$ is a normalization constant chosen so that the inner product of the eigenvector (41) and adjoint eigenvector (42) satisfy $\langle q^{*},q \rangle =1$.

### The second- and third-order terms

The formulas for the second- and third-order terms in Eq. (39) are given by

43 $$\begin{aligned}& \mathcal{B}(Y_{n},Y_{n}) = \bigl(b_{0}(Y_{n},Y_{n}),0,0, \ldots ,0\bigr)^{T}, \end{aligned}$$

44 $$\begin{aligned}& \mathcal{C}(Y_{n},Y_{n},Y_{n}) = \bigl(c_{0}(Y_{n},Y_{n},Y_{n}),0,0, \ldots ,0\bigr)^{T}, \end{aligned}$$

where the $b_{0}$ and $c_{0}$ terms are second- and third-order partial derivatives, respectively, of the right-hand sides of the system of $m+1$ first-order nonlinear difference equations corresponding to the nonlinear difference equations in Table 5. The values of the $b_{0}$ and $c_{0}$ terms for the five versions of the HIV and ELM models are shown in Table 6.

**Table 6:** Table of $b_{0}(Y_{n},Y_{n})$ and $c_{0}(Y_{n},Y_{n},Y_{n})$ formulas for HIV and ELM models

$b_{0}(Y_{n},Y_{n})$	HIV	ELM *Λ* = *β* − *r*, $\nu =\frac{\beta }{K} (\frac{\varLambda }{\beta })^{\frac{\gamma -1}{\gamma }}$
dnn	$-2\varepsilon h_{m} \tau y_{n} y_{n}$	$-(\gamma ^{2}+\gamma )\nu h_{m} \tau y_{n} y_{n}$
nnd, dnd	$-\varepsilon h_{m} \tau (y_{n} y_{n-m}+y_{n-m}y_{n})$	$\begin{array}[t]{l}{-}\nu h_{m} \tau [(\gamma ^{2}-\gamma ) y_{n-m}y_{n-m} \\ \quad {}+\gamma (y_{n}y_{n-m}+y_{n-m}y_{n})]\end{array}$
ndd, ddd	$-2\varepsilon h_{m} \tau y_{n-m} y_{n-m}$	$-\nu h_{m} \tau (\gamma ^{2}+\gamma )y_{n-m}y_{n-m}$

$c_{0}(Y_{n},Y_{n},Y_{n})$	ELM *Λ* = *β* − *r*, $\xi =\frac{\beta }{K^{2}}(\frac{\varLambda }{\beta })^{\frac{\gamma -2}{\gamma }}$
dnn	$-(\gamma ^{3}-\gamma )\xi h_{m} \tau y_{n}y_{n}y_{n} $
nnd, dnd	$\begin{array}[t]{l}{-}\xi h_{m} \tau [(\gamma ^{3}-3\gamma ^{2}+2\gamma )y_{n-m} y_{n-m}y_{n-m} \\ \quad {}+(\gamma ^{2}-\gamma )(y_{n}y_{n-m}y_{n-m}+y_{n-m}y_{n}y_{n-m}+y_{n}y_{n-m}y_{n-m})] \end{array}$
ndd, ddd	$-\xi h_{m} \tau (\gamma ^{3}-\gamma )y_{n-m}y_{n-m}y_{n-m} $

Following the algorithms in Li [19] and Kuznetsov [24], we concentrate on the expression for the critical coefficient $c_{1} (\tau _{c} )$,

45 $$ c_{1} (\tau _{c} )= \frac{g_{20}g_{11} (1-2\lambda _{c} )}{ 2 (\lambda _{c}^{2}-\lambda _{c} )} + \frac{ \vert g_{11} \vert ^{2}}{1-\overline{\lambda _{c}}} + \frac{ \vert g_{02} \vert ^{2}}{2 (\lambda _{c}^{2}-\overline{\lambda _{c}} )} + \frac{g_{21}}{2}, $$

where

46 $$\begin{aligned}& g_{02} = \bigl\langle q^{*},\mathcal{B} (\overline{q},\overline{q} )\bigr\rangle = \overline{D}b_{0}( \overline{q},\overline{q}), \\& g_{11} = \bigl\langle q^{*},\mathcal{B} (q,\overline{q} ) \bigr\rangle = \overline{D}b_{0}(q,\overline{q}), \\& g_{20} = \bigl\langle q^{*},\mathcal{B} (q,q )\bigr\rangle = \overline{D}b_{0}(q,q), \\& \omega _{11} = \frac{b_{0} (q,\overline{q} )}{P_{1} (1 )} p (1 ) - \frac{\langle q^{*},\mathcal{B} (q,\overline{q} )\rangle }{1-\lambda _{c}}q - \frac{\langle \overline{q^{*}},\mathcal{B} (q,\overline{q} )\rangle }{1-\overline{\lambda _{c}}} \overline{q} \end{aligned}$$

47 $$\begin{aligned}& \hphantom{\omega _{11}} = b_{0}(q,\overline{q}) \biggl\{ \frac{1}{P_{1} (1 )}p(1) - \frac{\overline{D}}{1-e^{i\omega _{c}}}q - \frac{D}{1-e^{-i\omega _{c}}} \overline{q} \biggr\} , \\& \omega _{20} = \frac{b_{0} (q,q )}{P_{1} (\lambda ^{2}_{c} )} p \bigl(\lambda _{c}^{2} \bigr)- \frac{\langle q^{*},\mathcal{B} (q,q )\rangle }{\lambda _{c}^{2}-\lambda _{c}}q- \frac{\langle \overline{q^{*}},\mathcal{B} (q,q )\rangle }{\lambda _{c}^{2}-\overline{\lambda _{c}}} \overline{q} \\& \hphantom{\omega _{20}} = b_{0}(q,q) \biggl\{ \frac{1}{P_{1}(e^{2i\omega _{c}})}p\bigl(e^{2i\omega _{c}} \bigr) -\frac{\overline{D}}{e^{2i\omega _{c}}-e^{i\omega _{c}}}q- \frac{D}{e^{2i\omega _{c}}-e^{-i\omega _{c}}} \overline{q} \biggr\} , \\& g_{21} = \bigl\langle q^{*},\mathcal{B}(\overline{q}, \omega _{20})\bigr\rangle +2 \bigl\langle q^{*}, \mathcal{B}(q,\omega _{11})\bigr\rangle +\bigl\langle q^{*}, \mathcal{C}(q,q,\overline{q})\bigr\rangle \\& \hphantom{g_{21}} = \overline{D}b_{0}(\overline{q},\omega _{20})+2 \overline{D}b_{0}(q, \omega _{11})+\overline{D}c_{0}(q,q, \overline{q}) , \end{aligned}$$

and

48 $$ p(\lambda )=\bigl(\lambda ^{m},\lambda ^{m-1},\ldots,\lambda ,1\bigr)^{T}. $$

The formulas for the $b_{0}$, $c_{0}$ and inner products required to compute the terms in (46) are shown in Table 7. The values of the characteristic polynomial $P_{1}(\lambda )$ (Eq. (20)) required in (46) are $P_{1} (1 )=h_{m}\tau (\eta - \rho )$ and $P_{1} (e^{2i\omega _{c}} ) =e^{2i(m+1)\omega _{c}}-e^{2im \omega _{c}}+h_{m}\tau ( \eta - \rho e^{2im\omega _{c}})$. The values of $p(1)$ and $p(\lambda _{c}^{2})$ can be obtained from (48).

**Table 7:**
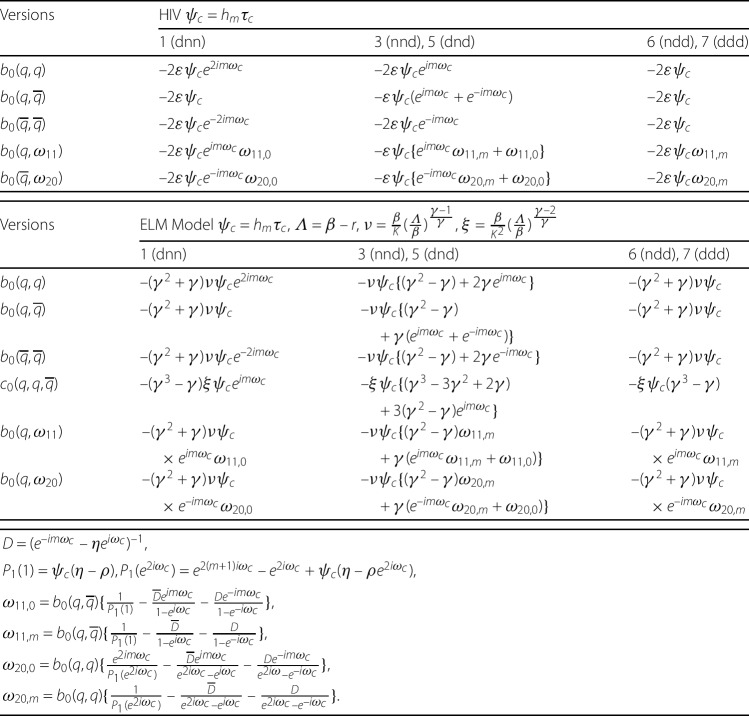
Formulas for the $b_{0}$, $c_{0}$ terms and inner products in the the critical constant $c_{1}(\tau _{c})$

## Numerical simulations

In this section, we present results of numerical simulations to illustrate the analytical results obtained in previous sections. We show the following.

1. The different behaviors of the five versions of the differential and difference equations which show bifurcations as the reproductive numbers and time delays are varied. In particular, we show the qualitatively different behavior of the supercritical limit cycles of the five versions.
2. The convergence of the Neimark–Sacker critical values to the Andronov–Hopf critical values as the value of *m* is increased.
3. The applications of the HIV models to HIV control and of the ELM models to the study of animal populations.

The parameter values used in the HIV simulations are given in Table 8 and were selected from [46]. The parameter values used in the ELM simulations are shown in Table 9 and were selected from [1].

**Table 8:** Values of parameters used for numerical simulation of HIV model

Parameter name	*B*	*α*	$p_{0}$	$\beta _{0}$	*C*	*δ*	*ε*
Set 1	0.005	0.01	0.1	0.5	0.5	0.0045	0.24
Set 2 [46]	0.05	0.05	0.01	0.5	0.5	0.0495	0.2
Set 3	0.05	0.05	0.0035	0.175	0.5	0.0498	0.0375

**Table 9:** Values of parameters used for numerical simulation of ELM model [1]

Parameter name	*r*	*β*	*K*	*γ*
Used values	3	4	4	2

### Comparison of qualitative behavior of versions

As shown previously, the equilibrium populations and values of $R_{0}$ for the differential equation and difference equation models are the same. The equilibrium population and $R_{0}$ values for the data sets in Tables 8 and 9 are shown in Table 10.

**Table 10:** Values of equilibrium points and $R_{0}$ of HIV and ELM models from Tables 8 and 9

Parameters	Disease-free	Endemic	$R_{0}$
HIV	$x^{*}_{0}=w^{*}_{0}$	$x^{*}_{1}=w^{*}_{1}=1-\frac{\delta }{\varepsilon }$	$\frac{\varepsilon }{\delta }$
Set 1	0	0.9813	53.333
Set 2	0	0.7525	4.0404
Set 3	0	–	0.7526
ELM	$x^{*}_{0}=w^{*}_{0}$	$x^{*}_{1}=w^{*}_{1}=K (1-\frac{r}{\beta } )^{\frac{1}{\gamma }}$	$\frac{\beta }{r}$
Set 1	0	2	1.333

Therefore, for zero time delay for both the differential equation and the difference equation models, the disease-free equilibrium is locally asymptotically stable for HIV set 3 of Table 8 and the endemic equilibria exist and are locally asymptotically stable for HIV sets 1 and 2 of Table 8 and ELM set 1 of Table 9.

### Andronov–Hopf and Neimark–Sacker bifurcations

For the parameter values in Tables 8 and 9, critical delay conditions for Andronov–Hopf bifurcation (Theorem 1) and Neimark–Sacker bifurcation (Theorem 3) of the endemic equilibria for the five HIV and ELM versions are shown in Tables 11 and 12. It can be seen that the results for the HIV and ELM models are similar. For the HIV model, the Andronov–Hopf and Neimark–Sacker bifurcations occur for the bounded region $1 < R_{0} < 3$ for version 1, and for unbounded regions $R_{0} > 1$ for versions 3, 5, 7, and for $R_{0} >3$ for version 6. Similarly, for the ELM model, the Andronov–Hopf and Neimark–Sacker bifurcations occur for the bounded region $1 < R_{0} < 2$ for version 1, and for unbounded regions $R_{0} > 1$ for versions 3, 5, 7, and for $R_{0} >2$ for version 6. It can also be seen from these tables, that, for version 1 of both models, the C5 conditions of Theorem 3 correspond to a repelling invariant closed curve for $\tau > \tau _{c}$, whereas for versions 3, 5, 6, 7 the C5 conditions correspond to an attracting invariant closed curve for $\tau > \tau _{c}$.

**Table 11:** Critical bifurcation values for endemic equilibrium of the HIV models

Version		1(dnn)		3(nnd), 7(ddd)	5(dnd)	6(ndd)
Range of $R_{0}$		$1 < R_{0} < 3$		$R_{0} > 1$	$R_{0} > 1$	$R_{0} > 3$
$R_{0}$		1.1	2.9	2.9	2.9	3.5
*ρ*		0.00405	−0.00405	0	0.0045	−0.0045
*η*		0.0045	0.0045	0.0086	0.0131	0.0068
*DDE*						
$\tau _{c}$		229.94	1371.68	183.72	99.492	457.26
$\phi _{c}$		1.962e−3	1.962e−3	8.550e−3	0.01225	5.031e−3
$\frac{d\mu }{d\tau } \vert _{\tau _{c}}$		1.848e−5	7.663e−8	2.108e−5	8.381e−5	1.729e−6
*Discrete*						
$\tau _{c}$	*m* = 22	229.58	1183.78	179.60	98.25	426.73
	*m* = 23	229.60	1191.27	179.78	98.31	428.01
$\omega _{c}$	*m* = 22	0.02005	0.1194	0.06981	0.05417	0.10219
	*m* = 23	0.01920	0.1143	0.06684	0.05187	0.09785
$\frac{dr}{d\tau } \vert _{\tau _{c}}$	*m* = 22	8.466e−6	5.153e−7	8.693e−6	1.763e−5	2.401e−6
	*m* = 23	7.760e−6	4.690e−7	7.959e−6	1.615e−5	2.193e−6
$\Re [e^{-i\omega _{c}}c_{1} (\tau _{c} ) ]$	*m* = 22	0.3454	0.6627	(3) −0.0371	−0.0222	−0.5919
				(7) −0.2868		
	*m* = 23	0.3317	0.6377	(3) −0.0355	−0.0212	−0.5667
				(7) −0.2745

**Table 12:** Critical bifurcation values for endemic equilibrium of the ELM models

Version		1(dnn)		3(nnd), 7(ddd)	5(dnd)	6(ndd)
Range of $R_{0}$		$1< R_{0}<2$		$R_{0}>1$	$R_{0}>1$	$R_{0}>2$
*γ* = 2		$1< R_{0}<1+\frac{2}{\gamma }$				$R_{0}>1+\frac{2}{\gamma}$
$R_{0}$		1.02	1.98	1.98	1.98	2.48
*ρ*		2.88	−2.88	0	3	−3
*η*		3	3	5.88	8.88	5.88
*DDE*						
$\tau _{c}$		0.3379	3.402	0.2671	0.1467	0.4165
$\phi _{c}$		0.8400	0.8400	5.880	8.358	5.057
$\frac{d\mu }{d\tau } \vert _{\tau _{c}}$		8.682	5.655e−3	9.971	38.45	2.693
*Discrete*						
$\tau _{c}$	*m* = 22	0.3376	2.6737	0.2612	0.14486	0.3958
	*m* = 23	0.3377	2.7012	0.2614	0.14494	0.3967
$\omega _{c}$	*m* = 22	0.0126	0.1267	0.0698	0.0545	0.0936
	*m* = 23	0.0121	0.1213	0.0668	0.0522	0.0896
$\frac{dr}{d\tau } \vert _{\tau _{c}}$	*m* = 22	0.0058	1.499e−4	0.0060	0.0119	0.0030
	*m* = 23	0.0053	1.358e−4	0.0055	0.0109	0.0028
$\Re [e^{-i\omega _{c}}c_{1} (\tau _{c} ) ]$	*m* = 22	0.6968	0.0889	(3) −0.0086	−0.0063	−0.0240
				(7) −0.0280		
	*m* = 23	0.6692	0.0855	(3) −0.0082	−0.0060	−0.0230
				(7) −0.0268		

**Figure 3:**
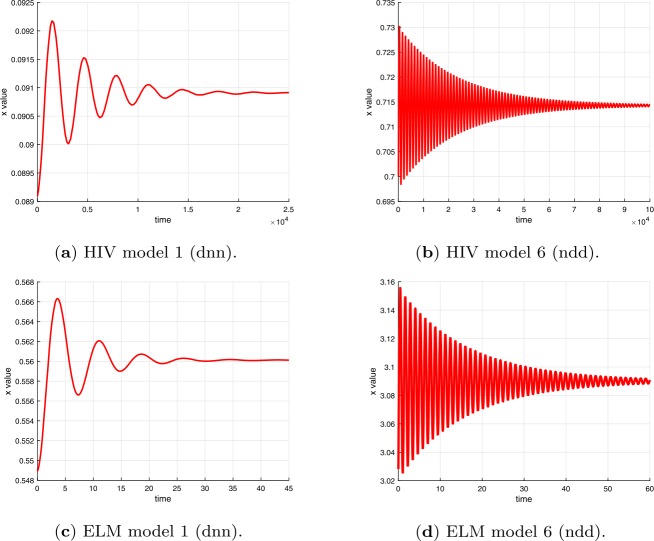
Solutions of HIV and ELM differential equations versions 1 and 6 for time delay less than critical delay $\tau < \tau _{c}$ (see Tables 11 and 12)

**Figure 4:**
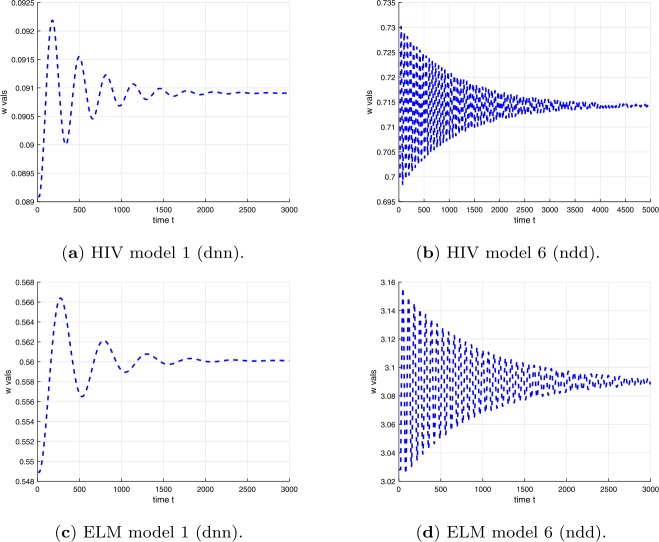
Solutions of HIV and ELM difference equations versions 1 and 6 for time delay less than critical delay at $m=22$ and $\tau < \tau _{c}$ (see Tables 11 and 12)

**Figure 5:**
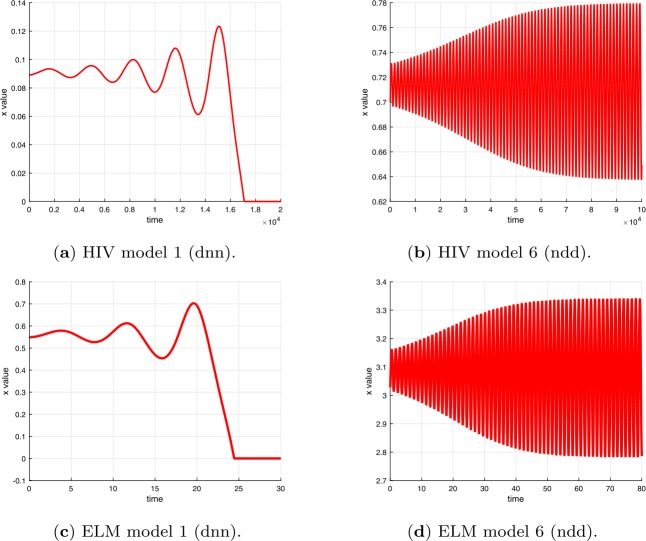
Solutions of HIV and ELM differential equations versions 1 and 6 for time delay greater than critical delay $\tau > \tau _{c}$ (see Table 11 and 12)

**Figure 6:**
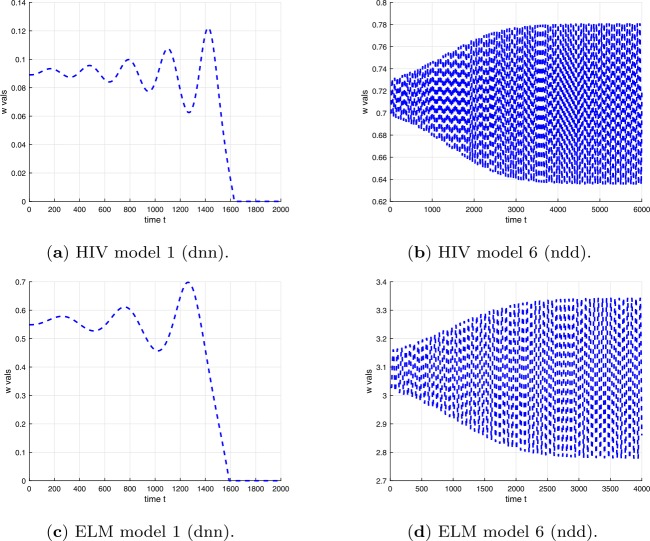
Solutions of HIV and ELM difference equations versions 1 and 6 for time delay greater than critical delay ($\tau > \tau _{c}$) at $m=22$ (see Tables 11 and 12)

Examples of plots of the time dependence of the solutions for time delays $\tau < \tau _{c}$ are shown in Fig. 3 for the differential equations in Table 1 and in Fig. 4 for the difference equations in Table 2. Examples of plots of the time dependence of the solutions for time delays $\tau > \tau _{c}$ are shown in Fig. 5 for the differential equations in Table 1 and in Fig. 6 for the difference equations in Table 2. The figures show plots for HIV and ELM versions 1 and 6. The plots for HIV and ELM models are qualitatively similar, but slightly different in detail. As noted in Sect. 8.2, the bifurcation occurs as *τ* increases through $\tau _{c}$ and the invariant closed curve is a repelling curve for version 1 and an attracting curve for version 6.

**Figure 7:**
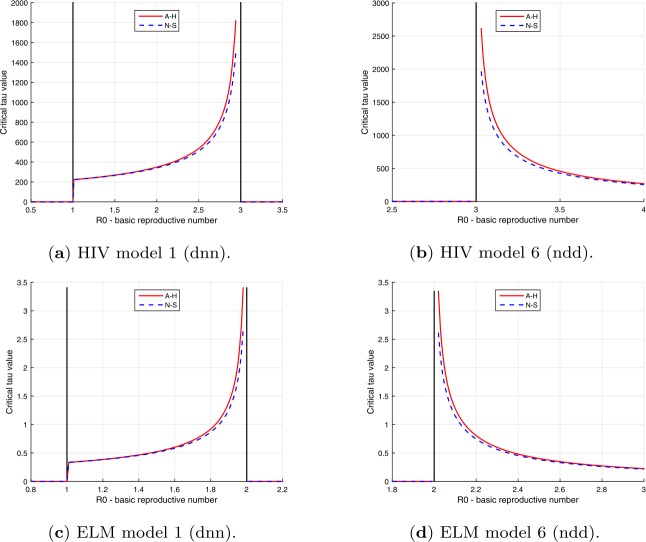
Plots of critical delays $\tau _{c}$ vs basic reproductive numbers $R_{0}$ for HIV and ELM versions 1 and 6

As shown in Tables 4, 11 and 12, Andronov-Hopf and Neimark-Sacker bifurcations can only occur for a range of $R_{0}$ values for the five versions with bifurcations. The plots in Fig. 7 give examples of the change in critical *τ* value with $R_{0}$ for version 1 (finite range) and version 6 (lower bound).

### Comparison of critical values for Andronov–Hopf and Neimark–Sacker bifurcations

From Tables 11 and 12, we have for condition (C3) of the Andronov–Hopf theorem that the values for the derivatives of the real part of the eigenvalues at the critical point $\frac{d\mu }{d\tau } \vert _{\tau _{c}}$ are greater than zero for all versions. Therefore, the Andronov–Hopf bifurcations will occur as *τ* increases through $\tau _{c}$ for all versions. It can also be seen from Tables 11 and 12 that case (1) of the condition (C5) of the Neimark–Sacker theorem occurs for version 1 (dnn) of the HIV and ELM discrete models and that case (2) occurs for versions 3 (nnd), 5 (dnd), 6 (ndd) and 7 (ddd). Therefore the Neimark–Sacker bifurcations will occur as *τ* increases through $\tau _{c}$ for all versions, but version 1 will have a repelling limit cycle and versions 3, 5, 6, 7 will have attracting limit cycles.

**Figure 8:**
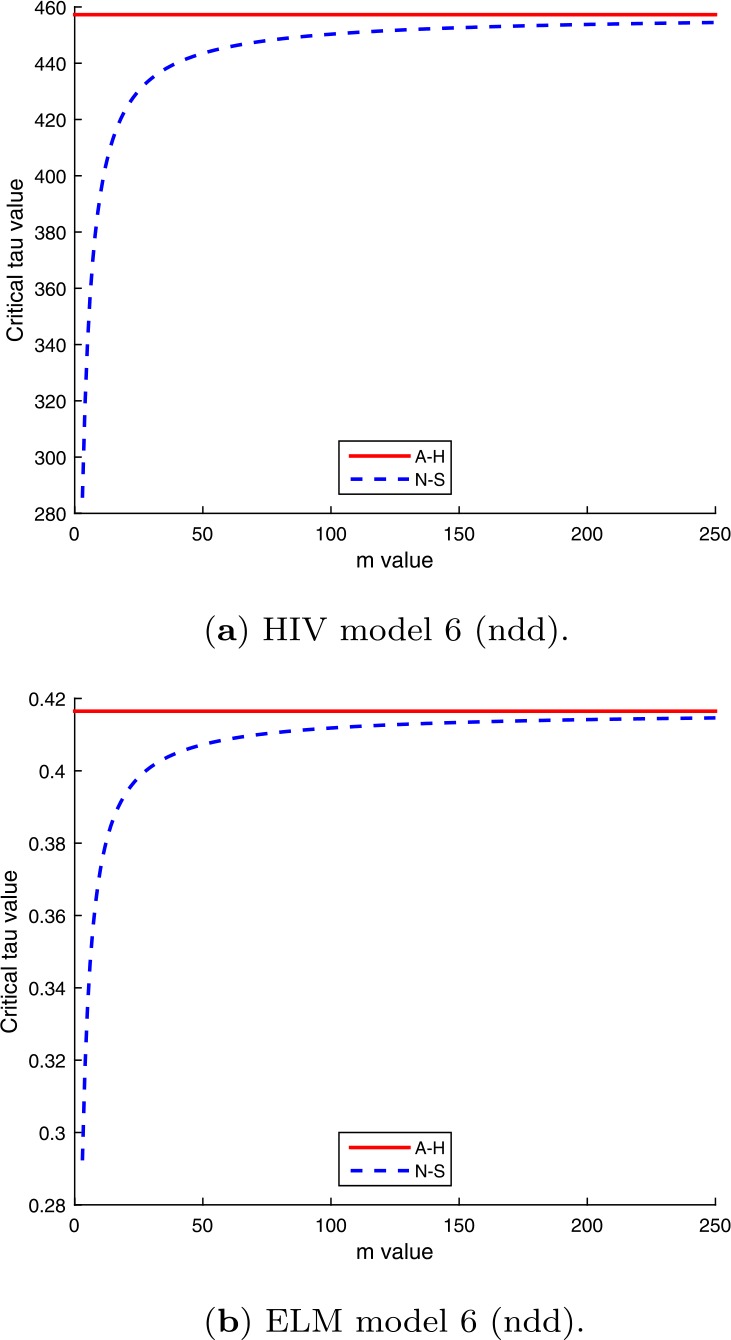
Plots showing convergence of Neimark–Sacker critical delay values for increasing *m* to Andronov–Hopf critical delay values for HIV and ELM version 6

A comparison of the critical delay values $\tau _{c}$ for the Andronov–Hopf and Neimark–Sacker bifurcations are shown in Fig. 8. It can be seen that the Neimark–Sacker values tend to the Andronov–Hopf values as the value of *m* increases, i.e., as the step size $h=1/m$ in the Euler difference equation approximation for the differential equation is reduced. These results suggest that difference equation models based on the Euler approximation can be used to obtain good estimates for critical delay values for models of the type studied in this paper.

### Effects of antiretroviral therapy

**Figure 9:**
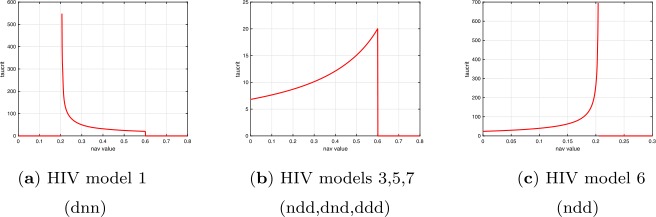
Plots of critical delay vs antiviral infectiousness factor

The effects of increasing the antiretroviral therapy factor $n_{\mathrm{av}}$ in (2) are shown in Fig. 9. Figure 9(a) shows the reduction in the basic reproduction number, Fig. 9(b) shows the effect on the equilibrium infected population and Fig. 9(c) shows the effect on the critical Andronov–Hopf bifurcation point. In practise, as stated in the introduction, it is well known (see, e.g., [29, 30]) that antiretroviral therapy cannot completely eliminate the virus. However, recent studies (see, e.g., [39, 41, 42]) have suggested that the therapy can reduce the virus sufficiently that HIV transmission from an HIV+ to an uninfected person will not occur.

An important idea in controlling diseases through vaccination and antiretroviral treatment is that of “herd immunity”, i.e., the level of immunity required to make the disease-free equilibrium stable and the endemic equilibrium negative. From Eqs. (2) and (7), we obtain

49 $$ R_{0}=\frac{\varepsilon }{\delta } = \frac{\beta _{0} (1-n_{\mathrm{av}})C-\alpha }{1-(1-n_{\mathrm{av}})p_{0} B} = 1 \quad \mbox{for } n_{\mathrm{av}}= 1-\frac{\alpha +B}{\beta _{0} C+p_{0} B}. $$

For sets 1 and 2 of Table 8, we have

50 $$ \mbox{set 1 :}\quad n_{\mathrm{av}} = 0.94, \qquad R_{0} = 53.33, \qquad \mbox{set 2 :}\quad n_{\mathrm{av}} = 0.60, \qquad R_{0}=4.04. $$

## Conclusions

In this paper, the effects of time delays and the associated Andronov–Hopf and Neimark–Sacker bifurcation properties have been studied for two one-dimensional models. The first model has been studied by previous authors as a model for HIV. This model includes the effects of vertical HIV transmission from mother to baby, the effects of births and deaths and treatment by antivirals. The second extended logistic growth model has been studied by previous authors as a model for population growth and for the life cycle of products and services. For each model, we have studied the dynamical behavior of a differential equation model and an equivalent difference equation model obtained from a forward Euler approximation.

For both HIV and ELM, we have shown that there are seven different time-delay versions of the differential equation and equivalent difference equation models and that the seven different versions have very different dynamical behavior. Five of these versions undergo bifurcations from the endemic equilibrium point and two of them have stable endemic equilibrium points for all values of time delay. We have given rigorous proofs of necessary and sufficient conditions for the existence and stability of the equilibrium points and for the existence of Andronov–Hopf and Neimark–Sacker bifurcations at critical values of the time delays for the five versions. For the Neimark–Sacker bifurcations, we have proved analytically and confirmed numerically that one version for each model has a supercritical repelling limit cycle and the remaining four versions have supercritical attracting limit cycles. We have also shown numerically that the same qualitative behavior occurs for the Andronov–Hopf bifurcations.

We have carried out numerical simulations for a range of biologically reasonable parameter values and obtained results that agree with the analytical results. The numerical results have shown that the equilibrium points and bifurcation behavior of an Euler approximation to a differential equation system have the same qualitative behavior as the differential equation system and that the critical bifurcation points of the difference equation converge to the critical times of the differential equation as the number of discretization points is increased.

The results of this paper show that a difference equation approximation can be used to study the dynamical behavior of time-delayed differential equation systems and to give reliable information about the existence and properties of bifurcations.
